# Warburg Effect Is a Cancer Immune Evasion Mechanism Against Macrophage Immunosurveillance

**DOI:** 10.3389/fimmu.2020.621757

**Published:** 2021-02-02

**Authors:** Jing Chen, Xu Cao, Bolei Li, Zhangchen Zhao, Siqi Chen, Seigmund W. T. Lai, Sabina A. Muend, Gianna K. Nossa, Lei Wang, Weihua Guo, Jian Ye, Peter P. Lee, Mingye Feng

**Affiliations:** ^1^ Department of Immuno-Oncology, Beckman Research Institute, City of Hope Comprehensive Cancer Center, Duarte, CA, United States; ^2^ Department of Biostatistics, University of Michigan School of Public Health, Ann Arbor, MI, United States

**Keywords:** macrophage, immunotherapy, phagocytosis, V-ATPase, microenvironment

## Abstract

Evasion of immunosurveillance is critical for cancer initiation and development. The expression of “don’t eat me” signals protects cancer cells from being phagocytosed by macrophages, and the blockade of such signals demonstrates therapeutic potential by restoring the susceptibility of cancer cells to macrophage-mediated phagocytosis. However, whether additional self-protective mechanisms play a role against macrophage surveillance remains unexplored. Here, we derived a macrophage-resistant cancer model from cells deficient in the expression of CD47, a major “don’t eat me” signal, *via* a macrophage selection assay. Comparative studies performed between the parental and resistant cells identified self-protective traits independent of CD47, which were examined with both pharmacological or genetic approaches in *in vitro* phagocytosis assays and *in vivo* tumor models for their roles in protecting against macrophage surveillance. Here we demonstrated that extracellular acidification resulting from glycolysis in cancer cells protected them against macrophage-mediated phagocytosis. The acidic tumor microenvironment resulted in direct inhibition of macrophage phagocytic ability and recruitment of weakly phagocytic macrophages. Targeting V-ATPase which transports excessive protons in cancer cells to acidify extracellular medium elicited a pro-phagocytic microenvironment with an increased ratio of M1-/M2-like macrophage populations, therefore inhibiting tumor development and metastasis. In addition, blockade of extracellular acidification enhanced cell surface exposure of CD71, targeting which by antibodies promoted cancer cell phagocytosis. Our results reveal that extracellular acidification due to the Warburg effect confers immune evasion ability on cancer cells. This previously unrecognized role highlights the components mediating the Warburg effect as potential targets for new immunotherapy harnessing the tumoricidal capabilities of macrophages.

## Introduction

In addition to malignant cells, the tumor microenvironment (TME) comprises a large population of resident-tissue cells and recruited immune cells (1–3). Tumor-associated macrophages (TAMs) are usually the most abundant groups of immune cells found within the TME (4–8). TAMs are generally classified into two major groups depending on their activation states: 1) classically activated macrophages (M1), and 2) alternatively activated macrophages (M2), both of which carry out different functions related to tumor development and angiogenesis (9), although these two populations are not always exclusive.

Recent breakthroughs in cancer immunology have identified a novel role of macrophages in recognizing cancer cells and attacking them *via* cellular engulfment. This process was termed “Programmed Cell Removal” (PrCR), during which cancer cells are directly phagocytosed by macrophages, bypassing the induction of cell death (10, 11). Oftentimes, in established tumors and metastases, cancer cells have evaded PrCR by developing self-protective mechanisms, among which the best known were the upregulation of “don’t eat me” signals to directly inhibit PrCR, such as CD47, MHCI and CD24 (12–15). Recent exciting progress demonstrated that PrCR may be induced by blocking “don’t eat me” pathways to remove this inhibitory effect, or by activating “eat me” pathways to enhance target cell recognition, therefore reinstating macrophage-mediated immunosurveillance and subsequently the elimination of cancer cells (16–19). CD47 has been identified as one of the most important anti-phagocytic “don’t eat me” signals, owing to its upregulation on many different types of human cancer cells (10, 11). Antibodies blocking the interaction between CD47 and its receptor on macrophages, signal regulatory protein alpha (SIRPα), have been shown to diminish the inhibitory signaling transduced to macrophages *via* the CD47-SIRPα axis, thus enabling the phagocytosis of cancer cells (16–19). PrCR induction has proven to be a promising new class of cancer immunotherapy in many preclinical cancer models, as well as clinical trials for hematopoietic malignancies and solid tumors (12, 13, 17, 18, 20–29).

However, the blockade of “don’t eat me” or induction of “eat me” signals usually were not sufficient to fully eradicate cancer cells. In addition to “don’t eat me” signals, it remains largely unexplored whether there are other self-protective mechanisms exploited by cancer cells to escape PrCR. The identification of such mechanisms may reveal novel therapeutic targets for inducing PrCR which can potentially be combined with existing immunotherapies to achieve a superior anti-cancer efficacy.

Distinct from that in normal tissue, the unique microenvironment in tumors directly impacts the metabolism, signaling and function of T cells, as revealed in previous studies in cancer immunology (30). The effects of tumor microenvironment on PrCR, however, remains largely unexplored. Glucose oxidation is one of the main sources of nutrients for cells and provides cells with energy in the form of ATP. Even in the presence of oxygen, cancer cells with high proliferation rates preferentially rely on glycolysis, an incomplete form of glucose oxidation, for energy production, a phenomenon known as the Warburg effect (31–33). While full oxidation of glucose produces carbon dioxide (CO_2_), aerobic glycolysis in cancer cells leads to the production of lactic acid in the form of lactate and protons. Cytosolic lactate is transported out of the cells *via* monocarboxylate transporters (MCT), while protons (H^+^) are secreted through membrane-bound transporters, leading to extracellular acidification (34, 35). Vacuolar ATPases (V-ATPase) are one of the most important H^+^ transporters responsible for maintaining the acidic extracellular pH (36, 37). V-ATPase is a multi-subunit protein complex, consisting of a membrane-anchored V0 domain, a cytosolic V1 domain, and accessory subunits. ATP hydrolysis in the V1 domain drives the rotation of the V0 domain for H^+^ translocation, acidifying the extracellular space (38). An acidic extracellular microenvironment has been shown to benefit cancer cells in their proliferation, survival, metastasis, and signal transduction (39–43), but whether it is involved in mediating the interaction between cancer cells and TAMs and regulating the immune evasion capabilities of cancers from PrCR has not been addressed.

Cancer progression has been positively correlated with the development of an immunosuppressive tumor microenvironment (44). Here, we demonstrate that extracellular acidification maintained by V-ATPase confers a self-protective mechanism on cancer cells independent of anti-PrCR mechanisms elicited by “don’t eat me” signals. This mode of defense occurs by attenuating the phagocytic ability of macrophages against the cancer cells. Therefore, targeting V-ATPase with pharmacological or gene editing tactics sensitizes cancer cells to macrophage-mediated immunosurveillance by restoring their susceptibility to PrCR, hampering tumor development. This approach synergizes with CD47 blocking, amplifying PrCR and yielding potent anti-cancer activity. In addition to this, we demonstrate that a blockade of the V-ATPase pathway in cancer cells promotes the expression of a cancer therapeutic target, CD71 (45), and thus can be used in combination with anti-CD71 antibodies to induce a “synthetic” PrCR. Our results reveal a previously unrecognized role of extracellular acidification, a common feature of many cancers, in generating an anti-PrCR immunosuppressive microenvironment, and may inspire future efforts for developing novel PrCR-based cancer immunotherapy for a variety of cancers.

## Materials and Methods

### Animals

BALB/c, NOD.Cg-Prkdc^scid^ Il2rg^tm1Wjl^/SzJ (NSG), and RAG2^−/−^ γc^−/−^ BALB/c mice were bred in the Animal Resources Center at City of Hope Comprehensive Cancer Center. BALB/c mouse strain was purchased from the Jackson Laboratory. RAG2^−/−^ γc^−/−^ mouse strain was a generous gift from Dr. Irving L. Weissman at Stanford University. All the procedures were approved by the Administrative Panel on Laboratory Animal Care at City of Hope Comprehensive Cancer Center (IACUC17051, approved in August 2017).

### Cell Culture

MDA-MB-231 and SW620 were cultured in DMEM (Gibco) supplemented with 10% FBS (Gibco) and 1% penicillin/streptomycin. DLD1, Raji and U937 cells were cultured in RPMI-1640 (Gibco) medium supplemented with 10% FBS (Gibco) and 1% penicillin/streptomycin. All cell lines were purchased from ATCC and maintained at 37°C in a 5% CO_2_ atmosphere. Cryopreservation of large quantities of low passage (below 3) cells was performed and cells with passage number below 20 were used in this study. Mycoplasma examination was routinely performed every two months.

### Generation of Macrophages

To generate mouse bone marrow derived macrophages (BMDMs), bone marrow cells were harvested from the femur and tibia of 6–12 weeks old BALB/c mice. After the red blood cells were lysed by ACK buffer for 2 min, the bone marrow cells were filtered through a 70-μm strainer, washed twice with DMEM medium supplemented with 10% FBS and cultured in IMDM medium supplemented with 10% FBS and 10ng/ml of MCSF for 6–8 days to differentiate into macrophages.

To generate human peripheral blood mononuclear cells (hPBMC) derived macrophages, human peripheral blood was obtained from the blood center at City of Hope Helford Clinical Research Hospital. CD14^+^ monocytes were enriched by Magnetic-activated Cell Sorting (MACS) with CD14 MicroBeads (Miltenyi Biotec) and subsequently cultured in IMDM with 10% human serum (Omega) for 6–8 days.

### Bioinformatics Analysis

V-ATPase related gene expression data of normal tissue and primary tumors from TCGA breast datasets were obtained from https://portal.gdc.cancer.gov/. In total, 10 ATPase H+ Transporting V0 subunit protein coding genes, 13 ATPase H+ Transporting V1 subunit protein coding genes and 3 ATPase H+ Transporting accessory protein coding genes were included into the analysis.

The correlation between expression of V-ATPase related genes including ATP6V1A, ATP6V0D1, ATP6AP1 and ATP6AP2 and M1/M2 macrophage infiltration from TCGA breast and colon cancer dataset was analyzed *via* TIMER2.0 (46). Gene expression datasets containing cancer patient outcomes were analyzed *via* PRECOG program (47) to assess the correlation between the expression of V-ATPase subunits and survival outcomes of patients with breast cancer, colon cancer, gastric cancer, lung cancer, pancreatic cancer, brain cancer and hematopoietic malignancies.

### Enrichment of PrCR Resistant Cells

A CD47^KO^ SW620 stable line was generated with Transcription activator-like effector nucleases (TALENs), as described previously (23). Briefly, SW620 cells were transfected with constructs containing TALEN pairs TGTCGTCATTCCATGCTTTG and TATACTTCAGTAGTGTTTTG which were designed to target exon2 of CD47. Cells were stained with anti-CD47 or isotype antibodies and CD47^-^ cells were sorted. CD47^KO^ SW620 cells was cocultured with BMDMs at a 1:1 ratio for 24 h. Surviving cells were detached from the cell culture plate by a brief incubation with trypsin during which majority of the BMDMs were still attached to the plate. The cells were then washed once with PBS and put back to cell culture plate with fresh growth medium (DMEM supplemented with 10% FBS) for a recovery of 2–3 days. Cell numbers were determined by counting GFP+ cells under a fluorescent microscope. The cells were then added to freshly prepared BMDMs at a 1:1 ratio to repeat the phagocytosis selection. After 10 rounds of such selection process, the derived CD47^KO^ SW620 sub-line cells (termed P10) were sorted and cultured in fresh growth medium for a recovery of 7 days. CD47^KO^ SW620 cells that were cultured for the same procedure and time period but in the absence of BMDMs were used as the control line (termed P0).

The expression of CD47 on P0 and P10 cells was examined by flow cytometry with anti-CD47 antibody (clone B6H12, BD Biosciences) to ensure no CD47-expressing cells were enriched. Resistance of P10 cells to PrCR was evaluated by a phagocytosis assay.

### Extracellular pH, Lactic Acid, and V-ATPase Activity Measurement

To measure extracellular pH, SW620, DLD1 or MDA-MB-231 cells were seeded into the 6-well plates with 5x10^5^ cells per well, including cells treated with vehicles or concanamycin (10nM), or cells transduced with Cas9 and control sgRNAs (non-targeting) or sgRNAs targeting ATP6AP2, as indicated in the figure legend, and pH of culture medium was measured at the indicated time points by pH meter (ThermoFisher).

To measure lactic acid secretion, SW620 P0 or P10 cells were seeded into the 24-well plates with 10^5^ cells per well and cultured with DMEM medium supplemented with 10% dialyzed FBS (Gibco). Culture media of P0 and P10 were collected at 24, 48, and 72h for lactate measurement. Cell numbers were also recorded at these time points. The lactate release was measured by L-Lactate Assay Kit (Eton Bioscience).

To measure V-ATPase activity, SW620 P0 or P10 cells were seeded into the 6-well plates with 5x10^5^ cells per well. After overnight incubation, fresh culture medium with 100 nmol/L Lysotracker Red DND-99 (ThermoFisher) were added to the cells and incubated at 37°C in a 5% CO2 atmosphere for 50 min. Cells were washed once with PBS and incubated with fresh medium for another 10 min before the imaging. Images of brightfield and fluorescence were taken by Cytation 3 (BioTek). The mean (Texas Red 586,647) fluorescent intensity was calculated by Cytation 3 and set as the LysoTracker signal. The V-ATPase activity was quantified by the LysoTracker signal per cell.

### CRISPR/Cas9-Mediated Gene Editing

Suppression of gene expression was performed using the CRISPR/Cas9 system in SW620, DLD1 and MDA-MB-231 cells. Pairs of primers containing control sgRNAs (non-targeting) or sgRNAs targeting human CD47, ATP6V1A, ATP6V1D, ATP6V0D1, ATP6AP1, or ATP6AP2 genes were designed and cloned into the all-in-one LentiCRISPR V2 vector (48). The LentiCRISPR V2 vector was transfected with the packing plasmids into HEK293T cells. Forty-eight hours after transfection, lentiviruses were collected and filtered through 0.45um filters to remove residual 293T cells and cell debris. SW620, DLD1 or MDA-MB-231 cells were cultured in 6-well cell culture plates with 2x10^5^ cells per well and incubated with lentiviruses for 48 h in the presence of polybrene (8µg/ml), recovered for 24 h and selected with puromycin (2µg/ml) for at least 6 days to obtain stable lines with indicated genes suppressed.

The following sgRNA sequences were used:

Control 1 (Non-targeting): GAACGUAGAAAUUCCCAUUU (48)
Control 2 (LacZ): UUGGGAAGGGCGAUCGGUGC (49)
Human CD47: CUACUGAAGUAUACGUAAAG (48)
Human ATP6V1A: UGGAGAGAUUAUUCGAUUGG (48)
Human ATP6V1D: CUGUCGAAAUCGAAGAGUUA (48)
Human ATP6V0D1: AGUCAUCGAUGACCGGCUCA (48)
Human ATP6AP1: UGGUGCUUCCUGCCGUCGAC (48)
Human ATP6AP2: AGGAGAGCGGAUCCCAGACG (48)

For the rescue experiment, sgRNA targeting ATP6AP2 was cloned into the all-in-one LentiCRISPR V2-Blast vector. LentiCRISPR v2-Blast was a gift from Dr. Mohan Babu (Addgene plasmid #83480). ATP6AP2 cDNA with synonymous mutations was cloned into pCDH vector (System Biosciences) with a HA tag (TATCCTTACGACGTGCCTGACTACGCC) to its C-terminus. The targeting sequence by sgRNA and PAM sequence in ATP6AP2 was mutated from AGGAGAGCGGATCCCAGACGTGG to AGGTGAACGCATTCCTGATGTAG by site-directed mutagenesis and confirmed by Sanger sequencing. The LentiCRISPR V2-blast vector and pCDH vector were transfected with the packing plasmids into HEK293T cells, respectively. Forty-eight hours after transfection, lentiviruses were collected and filtered through 0.45um filters to remove residual 293T cells and cell debris. SW620 cells were cultured in 6-well cell culture plates with 2x10^5^ cells per well and incubated with lentiviruses generated from LentiCRISPR V2 and pCDH for 48 h in the presence of polybrene (8µg/ml), recovered for 24 h and selected with blasticidin (30µg/ml) and puromycin (2µg/ml) for at least 2 days to obtain stable lines with suppression and/or exogenous expression of ATP6AP2.

### Flow Cytometry Analysis

Anti-human CD47 (clone B6H12, BD Biosciences), anti-mouse F4/80 (clone BM8, BioLegend), anti-Sirpα (clone P84, BioLegend), anti-mouse/human CD11b (clone M1/70, BioLegend), anti-mouse CD45 (clone 30-F11, BioLegend), anti-mouse MHC II (clone M5/114.15.2, BioLegend), anti-mouse CD206 (clone C068C2, BioLegend), anti-mouse CD80 (clone 16-10A1, BioLegend), anti-mouse CD86 (clone GL-1, BioLegend), anti-mouse PD-L1 (clone 10F.9G2, BioLegend), anti-mouse Gr1(clone RB6-8C5,BioLegend), anti-human CD14 (clone HCD14, BioLegend), and anti-human CD71 (clone CY1G4, BioLegend; clone OKT9, ThermoFisher; clone L01.1 and clone M-A712, BD Biosciences) were used for FACS analyses. Antibodies were Phycoerythrin (PE)-, PE/Cyanine7, APC, APC/Cyanine7, PerCP/Cyanine5.5, PE/Dazzle™ 594, Alexa Flour^®^ 700 or BV605 conjugated, or fluorophore-conjugated secondary antibodies were used. Annexin V (BD Biosciences), Sytox blue (ThermoFisher), 7-Aminoactinomycin D (7-AAD, ThermoFisher), or Zombie Violet™ Fixable Viability Kit (Biolegend) was used to exclude dead cells. Flow cytometry was performed using the BD LSRFortessa cell analyzers (BD).

### Phagocytosis Assay

Phagocytosis assays were performed with mouse bone marrow-derived macrophages (BMDMs) or human peripheral blood monocyte-derived macrophages.

For short-term phagocytosis assays, macrophages and target cancer cells were detached from cell culture plates by incubation with TrypLE (Gibco). Cells were washed twice with PBS and cell numbers were determined by cell counting. For each sample, 0.1x10^6^ BMDMs or hPBMC macrophages were cocultured with 0.2x10^6^ cancer cells expressing a GFP-luciferase fusion protein in IMDM medium at 37°C in the cell culture incubator for 2 h in the presence of CD47-blocking antibodies or other agents as indicated in the figure legend. Cells were washed once with FACS buffer (PBS supplemented with 2% FBS). Mouse macrophages were stained with anti-mouse F4/80 antibody (clone BM8, BioLegend) conjugated with PE-Cy7 and hPBMC macrophages were stained with anti-human CD14 antibody (clone HCD14, BioLegend) conjugated with APC. Phagocytosis index was determined by the percentage of macrophages that have phagocytosed cancer cells, thus becoming double positive (PE-Cy7^+^GFP^+^ or APC^+^GFP^+^) among the entire macrophage population (PE-Cy7^+^ or APC^+^) by flow cytometry analyses using the BD LSRFortessa cell analyzers. Anti-CD47 antibody (clone B6H12, BioXCell) or anti-Sirpα (clone P84, BioLegend) antibody was used in phagocytosis assays for blocking CD47-Sirpα interaction. Phagocytosis index was normalized to the maximal response by each independent donor (human or mouse) against each cell line.

In some phagocytosis assays as indicated, cancer cells were labeled with pHrodo™ Red succinimidyl ester (Thermo). SW620 cells were washed twice with PBS and resuspended with PBS at 0.2 million cells/ml. The pHrodo™ Red was added to a final concentration of 120 ng/ml. Cells were incubated with pHrodo™ Red for 30 min at room temperature in the dark. Cells were washed twice with PBS and cell numbers were determined by cell counting. For each sample, 0.1x10^6^ BMDMs or hPBMC macrophages were cocultured with 0.2x10^6^ cancer cells expressing a GFP-luciferase fusion protein in IMDM medium at 37°C in the cell culture incubator for 2 h in the presence of CD47-blocking antibodies or other agents as indicated in the figure legend. The pHrodo™ Red dye becomes brightly red in phagosomes, indicating the labeled cells were engulfed by macrophages.

For long-term phagocytosis assays, 0.1x10^6^ macrophages and target cancer cells expressing a GFP-luciferase fusion protein were cocultured in 96-well cell culture plate at ratios of 1:1 to 1:3 for 24 h. Samples with cancer cells alone (without macrophages) were used as controls. The plates were washed once with PBS and luciferin (SYDlabs) was added to the wells. Surviving cancer cells were quantified by reading the luminescence signals with Cytation 3. Cancer cell killing was determined by normalizing the luminescence of surviving cancer cells to that of the same type of cancer cells with the same treatment but in the absence of macrophages.

### Western Blotting

The ATP6AP2 gene knockdown efficiency was examined by western blotting. Cells were lysed in lysis buffer (PBS supplemented with 0.5% SDS) along with protease inhibitors and protein concentrations were quantified by BCA protein assay. Protein samples were diluted in the loading buffer (62.5 mM Tris-HCl pH 6.8, 10% Glycerol, 2% SDS, 0.01% Bromophenol Blue, 100 mM DTT) and subjected to SDS-polyacrylamide gel electrophoresis. For immunoblotting, anti-HA (Covance), anti-ATP6AP2 (Novus Biologicals) and anti-GAPDH (Cell Signaling, loading control) primary antibodies and HRP-conjugated anti-rabbit IgG or anti-mouse IgG secondary antibodies were used.

### *In Vivo* Tumor Models

RAG2^−/−^ γc^−/−^ mice were engrafted with SW620, DLD1 or MDA-MB-231 cells expressing a GFP-luciferase fusion protein to evaluate the role of V-ATPase-mediated extracellular acidification in tumor development and metastasis. For the SW620 and DLD1 models, 0.05x10^6^ Ctrl^KD^ (sgRNA targeting LacZ gene) or ATP6AP2^KD^ cells were suspended in RPMI medium with 25% Matrigel Matrix (Corning) and subcutaneously injected into the 6-8 weeks old RAG2^−/−^ γc^−/−^ mice. Mice were treated with PBS (control) or CD47-blocking antibody (clone B6H12, BioXCell) once per week starting 7 days after tumor engraftment. CD47-blocking antibody was diluted in PBS and used as a dose of 2.5mg/kg body weight. For the MDA-MB-231 model, 0.2 x 10^6^ Ctrl^KD^ (sgRNA targeting LacZ gene), CD47^KD^, ATP6AP2^KD^, or Double^KD^ cells were suspended in DMEM medium with 25% Matrigel Matrix (Corning) and injected into the mammary fat pad of 6-8 weeks old female NSG mice. Bioluminescent imaging was used to monitor tumor development and metastasis. In detail, tumor-bearing mice were injected intraperitoneally with D-luciferin in PBS with a dose of 139mg luciferin/kg body weight. Imaging was performed using Lago X (Spectral Instruments Imaging) and bioluminescence signals were analyzed with Aura Image software (Spectral Instruments Imaging). All the tumor-bearing mice were sacrificed at the end point.

The tumors developed by Ctrl^KD^ and ATP6AP2^KD^ DLD1 cells were collected, minced into small pieces, and dissociated in DMEM with Liberase TM enzymes (ThermoFisher) and DNase (ThermoFisher) at 37°C until a single-cell suspension was achieved. Red blood cells were lysed with ACK lysis buffer. Cells from the tumor were washed twice with FACS buffer (PBS supplemented with 2% FBS) and filtered through a 70-um cell strainer to remove undigested pieces. FcR blocker (Miltenyi) was used to treat the cells to block non-specific binding of antibodies and the cells were then stained with indicated antibodies and subjected to flow cytometry analysis. A combination of cell surface markers (CD45^+^ F4/80^+^CD11b^+^Gr-1^-^) and Zombie Violet ^-^ were used to define tumor-associated macrophages (TAMs). The expression of CD206, MHC II, CD80, CD86, PD-L1 on TAMs was examined.

### Quantitative PCR

Total RNA of BMDM cultured under pH 7.4 and 6.5 for 48h was extracted with an RNA extraction kit (Qiagen). cDNA was generated by SuperScript™ VILO™ cDNA Synthesis Kit (Invitrogen) and quantitative PCR was performed with SYBR Green (BIOLINE) and acquired by CXF96 real-time system (BIO-RAD).

The following primer sequences were used:

Gapdh F: CCAGTTGGTAACAATGCCATGT
Gapdh R: GAGTTGCTGTTGAAGTCGCA
Cxcl10 F: CCAAGTGCTGCCGTCATTTTC
Cxcl10 R: GGCTCGCAGGGATGATTTCAA
Tnf F: CTGAACTTCGGGGTGATCGG
Tnf R: GGCTTGTCACTCGAATTTTGAGA
Ccl5 F: GCTGCTTTGCCTACCTCTCC
Ccl5 R: TCGAGTGACAAACACGACTGC
Arg1 F: CTCCAAGCCAAAGTCCTTAGAG
Arg1 R: AGGAGCTGTCATTAGGGACATC
Fn1 F: ATGTGGACCCCTCCTGATAGT
Fn1 R: GCCCAGTGATTTCAGCAAAGG

### Cytokine/Chemokine Analysis

Ctrl^KD^ and ATP6AP2^KD^ MDA-MB-231 cells were seeded in the 12-well plates at a density of 5x 10^5^ cells per well for 48 h. Cell culture medium from these two lines were collected and centrifuged. The supernatants were collected and submitted to Eve Technologies for Human Cytokine Array/Chemokine Array 48-plex discovery arrays which were analyzed using Millipore MILLIPLEX human cytokine/chemokine kit.

### Antibody Array

Cell surface proteome on cancer cells with V-ATPase blockade was examined by a flow cytometry-based high-throughput antibody array.

LEGENDScreen kit purchased from BioLegend contained 332 PE-conjugated monoclonal antibodies against human cell surface proteins. The kit also contains 10 mouse, rat and hamster Ig isotype controls. Lyophilized antibodies in 96-well plates were reconstituted with deionized water. SW620 cells treated with vehicles or concanamycin (10nM) overnight were detached from the cell culture dishes by trypsin and cell numbers were determined by cell counting. Cells were washed twice with cell staining buffer included in the kit and seeded to ultra-low attachment 96-well plate with 10^5^ cells per well using multi-channel pipettes. Reconstituted antibodies were added to each well of cells and gently mixed with the cell suspensions. The plates were then incubated at 4^°^C for 30 min, washed twice with cell staining buffer and subjected to flow cytometry analyses using the High Throughput Sampler (HTS) mode of BD LSRFortessa cell analyzers.

### Statistical Analysis

Statistical analyses were performed under GraphPad Prism 8.3.0 (GraphPad Software) and Excel (Microsoft). Data are presented as mean ± SD. Student *t* tests for two group comparisons and one-way *ANOVA* test for multi-group comparisons were used for the study. A *p*-value of <0.05 was considered statistically significant.

## Results

**Figure 1:**
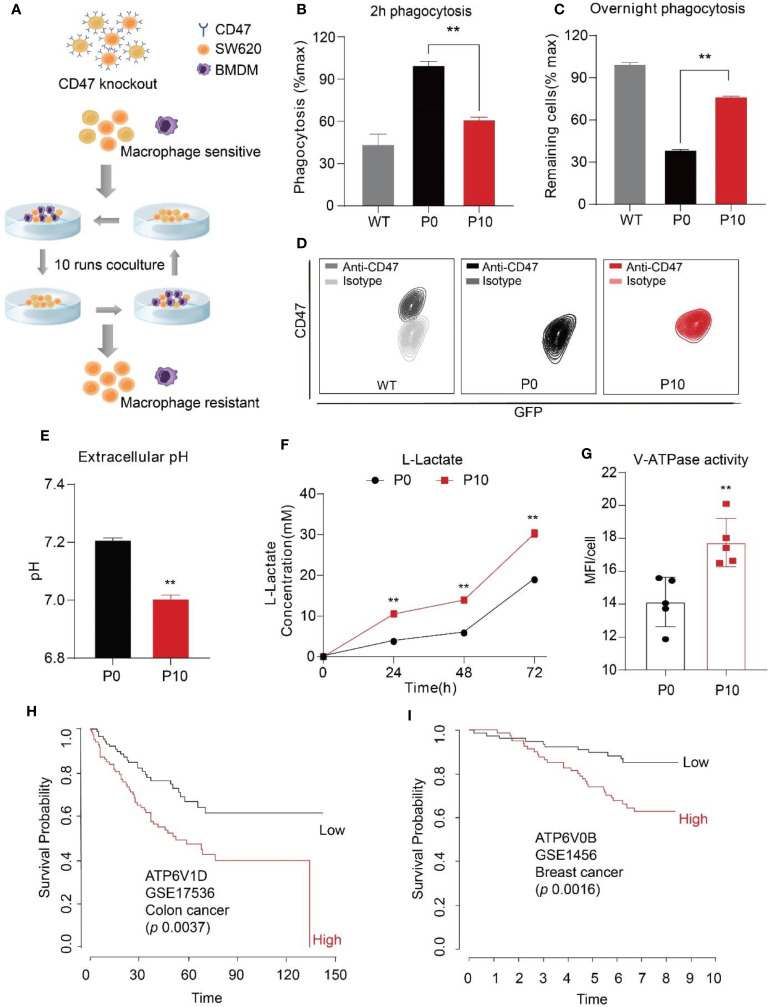
Increased extracellular acidification induced by cancer cells correlated with resistance to macrophage-mediated PrCR and worse prognosis in patients. **(A)** A Schematic showing the strategy to generate cells resistant to macrophage-mediated PrCR. **(B, C)** A 2h phagocytosis assay **(B)** and an overnight phagocytosis assay **(C)** demonstrated the resistance of P10 cells to macrophage-mediated PrCR. P10 group was compared with the P0 group. (Normalization is described in details in “Methods”. For overnight assay, the values of WT cells were normalized as 100%) **P < 0.01 (*t* test). **(D)** Flow cytometry analysis of CD47 expression on SW620 WT, P0 and P10 cells. **(E)** P10 cells acidified extracellular microenvironment more rapidly than P0 cells. P10 group was compared with the P0 group, 72h culture medium was collected for analysis. **P < 0.01 (*t* test). **(F)** A higher concentration of L-lactate was detected in the extracellular medium of P10 cells, as compared to that of P0 cells. P10 group was compared with the P0 group at individual time point. **P < 0.01 (*t* test). **(G)** Lysotracker staining indicated an increased activity of V-ATPase in P10 cell, as compared to that in P0 cells. P10 group was compared with the P0 group. **P < 0.01 (*t* test). **(H, I)** Upregulation of V-ATPase subunits V1D1 **(H)**, V0B **(I)**] correlates with a worse overall survival rate in patients with colon cancer and breast cancer.

To identify mechanisms exploited by cancer cells against PrCR beyond CD47 upregulation, we first knocked out CD47 expression in SW620, a human colon cancer line, by transducing the cells with TALEN (transcription activator-like effector nucleases) plasmids and sorting for CD47 negative cells. A phagocytosis assay was then performed to examine the susceptibility of cancer cells to PrCR by macrophages. In most cancers, tumor-associated macrophages (TAMs) originated from bone marrow progenitor cells and monocytes, which were recruited to tumors and differentiated into TAMs. Therefore, bone marrow-derived macrophages (BMDMs) have been established as a sound research tool for assessing the phagocytic ability of macrophages against cancer cells. Next, we designed a PrCR selection assay to enrich for cells resistant to PrCR (**Figure 1A**). In this assay, CD47^KO^ SW620 cells were co-cultured with BMDMs for 24 h. Surviving SW620 cells were collected from the tissue culture plates and subjected to another round of co-culture with freshly prepared BMDMs. We reasoned that the repeated selection would lead to the depletion of cells susceptible to PrCR, and thus the enrichment of cells resistant to PrCR. After ten rounds of such selection, we collected the surviving SW620 CD47^KO^ cells (which we termed P10). We demonstrated that consistent with previous studies, the parental CD47^KO^ cells became much more vulnerable to macrophage phagocytosis due to the loss of CD47 protection (**Figures 1B, C** **)**, while the P10 cells surviving from PrCR selection had become significantly more resistant to PrCR, as compared to the parental cells (which we termed P0) (**Figures 1B, C**, and **Figures S1A, B**). We assessed phagocytosis with two independent approaches: 1) to examine the extent by which macrophages have phagocytosed cancer cells after 2 h of macrophage-SW620 coculture, or 2) to examine surviving cancer cells after an overnight macrophage-SW620 coculture (**Figures 1B, C** **)**. The same conclusions were achieved from both methods, indicating PrCR-resistant cells have been enriched in P10. Importantly, the P10 cells remained CD47 deficient (**Figure 1D** and **Figure S1C**), excluding the possibility that our selection had enriched residual CD47-expressing cells.

During glycolysis in cancer cells, lactic acid resulting from the Warburg effect is transported out of the cells to acidify the extracellular microenvironment (50). Interestingly, a significantly lower pH and higher concentration of lactate were detected in the extracellular medium taken from P10 cells compared to the parental cells (**Figures 1E, F** **)**, although the proliferation rate of P0 and P10 was comparable (**Figure S1D**), suggesting that the P10 cells had acidified the extracellular medium more rapidly. In addition, an increase in lysotracker staining was observed in P10 cells (**Figure 1G**), indicating enhanced assembly and activity of V-ATPases. V-ATPase is critical for maintaining an acidic pH within the lysosomal lumen for protein digestion and recycling (37, 38). Beyond this, V-ATPase also pumps protons out of the cells, leading to an acidic microenvironment in cancer tissues (51, 52). To assess the implications on clinical cases, we analyzed a large cohort of gene expression datasets containing cancer patient outcomes with the PRECOG program (47), including that of breast cancer, colon cancer, and hematopoietic malignancies. We found that a higher expression of V-ATPase subunits, including the V0 domain, V1 domain, and accessory domains, was correlated with a worse overall survival outcome (**Figures 1H, I** and **Figures S1E–S1J**). These data indicate that the hyper-activation of V-ATPase is clinically relevant to the progression of a wide range of cancers.

**Figure 2:**
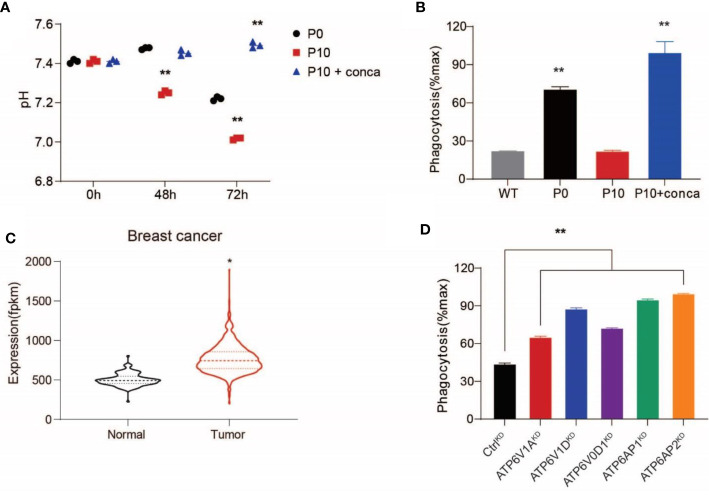
Upregulated V-ATPase activity conferred resistance to macrophage-mediated PrCR. **(A)** Pharmacological inhibition of V-ATPase with concanamycin A (10 nM) diminished extracellular acidification of P10 cells. **P < 0.01 (one-way *ANOVA* test). **(B)** A phagocytosis assay with BMDMs showing that concanamycin A treatment restored the vulnerability of P10 cells to macrophage-mediated PrCR. Each group was compared with the WT group. **P < 0.01 (one-way *ANOVA* test). **(C)** Significant up-regulation of V-ATPase subunit coding genes in breast cancer (n=1,102) tissues as compared to the adjacent normal tissues, based on TCGA datasets (In total 10 ATPase H^+^ Transporting V0 subunit protein coding genes, 13 ATPase H^+^ Transporting V1 subunit protein coding genes and 3 ATPase H^+^ Transporting accessory protein coding genes were included into the analysis). *P < 0.05 (*t* test). **(D)** A phagocytosis assay with BMDMs showing that knocking-down the expression of the individual subunit of V-ATPase with CRISPR increased the susceptibility of SW620 cells to PrCR. Each group was compared with the Ctrl^KD^ group. **P < 0.01 (one-way *ANOVA* test).

Because the evasion of PrCR has been shown to be an important mechanism contributing to worse prognosis (53), we decided to examine whether V-ATPase plays a role in inhibiting macrophage-mediated PrCR. First, we used pharmacological inhibitors to block V-ATPase activity in SW620 cells. We found that treating the cells with concanamycin A, which blocks the ATP-binding site in the V1 domain to disrupt its ATPase activity, diminished extracellular acidification (**Figure 2A**) and significantly promoted PrCR of P10 cells to a similar level of that of P0 cells (**Figure 2B**), indicating that V-ATPase-mediated self-protection is a major mechanism accounting for their resistance to PrCR. Previous studies have demonstrated that dysfunction in the subunits of the V0 domain, V1 domain, and accessory subunits AP1 or AP2 compromises the ability for V-ATPase to pump protons (54–56). Analyses of gene expression profiling of patient specimens from GDC TCGA Breast Cancer dataset revealed an enhanced expression of V-ATPase subunits in primary tumors as compared to normal tissues (**Figure 2C**). Therefore, we used a CRISPR/Cas-9-mediated gene editing approach to knock down the expression of these subunits individually in SW620 cells and evaluated their effects by examining SW620 susceptibility to macrophages. Enhanced PrCR was observed across the single subunit knockdowns (**Figure 2D**), indicating that a functional V-ATPase complex is critical for maintaining the anti-PrCR ability of cancer cells.

**Figure 3:**
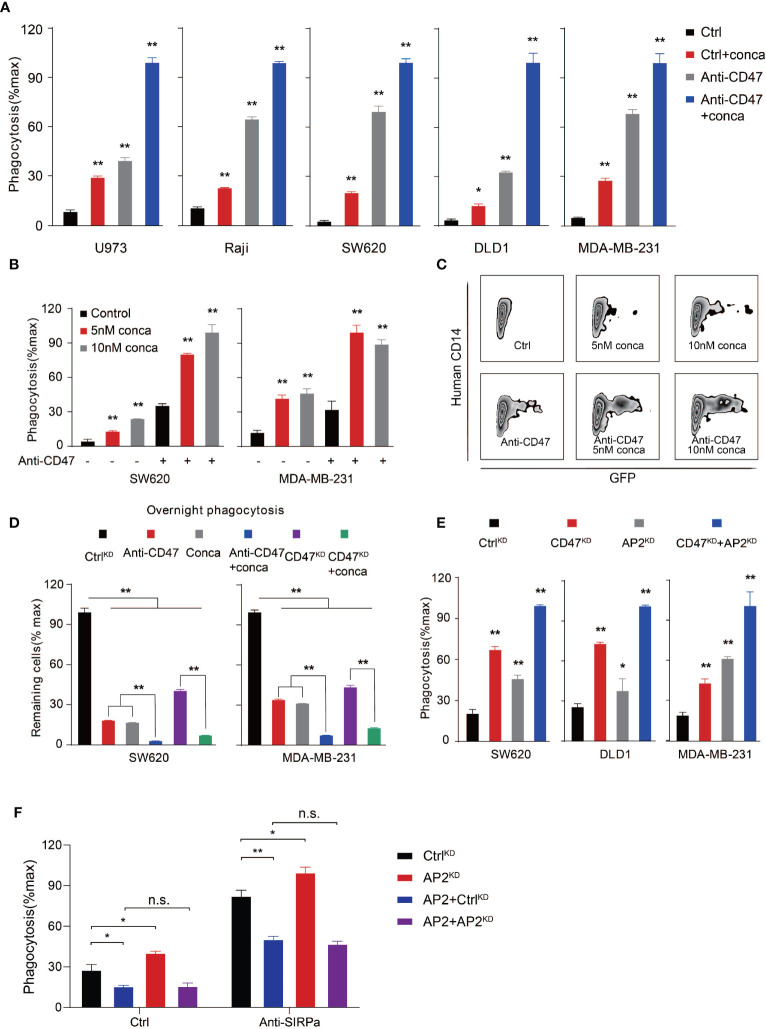
V-ATPase inhibition synergized with CD47 blockades to induce PrCR. **(A)** Phagocytosis assays with BMDMs showing that pharmacological inhibition of V-ATPase with concanamycin A (10 nM) promoted PrCR and increased CD47-blocking antibody-induced phagocytosis. The assays were performed with human lymphoma (U973 and Raji), colon cancer (SW620 and DLD1) and breast cancer (MDA-MB-231) lines. Each group was compared with the Ctrl group. *P < 0.05, **P < 0.01 (one-way *ANOVA* test). **(B)** A phagocytosis assay with human peripheral blood monocyte-derived macrophages showing that concanamycin A treatment enhanced PrCR of SW620 and MDA-MB-231 cells in the absence or presence of CD47 blocking antibodies. Each group was compared with corresponding non-treated or treated Ctrl group. **P < 0.01 (one-way *ANOVA* test). **(C)** Representative flow cytometry plots for **(B)** Macrophages were labeled with anti-CD14. SW620 cells were GFP+. Concanamycin A treatment increased the percentages of human macrophage phagocytosing cancer cells (hCD14+GFP+) with or without anti-CD47 antibody. **(D)** An overnight phagocytosis assay showing macrophage-mediated PrCR induced by either CD47 blocking antibody or genetic deletion of CD47 was largely potentiated by treatment of SW620 and MDA-MB-231 cells with concanamycin A (the values of Ctrl^KD^ were normalized as 100%). **P < 0.01 (one-way *ANOVA* test). **(E)** A phagocytosis assay with BMDMs showing that genetic knockdown of ATP6AP2 expression was able to induce PrCR of SW620, DLD1 and MDA-MB-231 cells and synergized with knockdown of CD47 expression. Each group was compared with the Ctrl^KD^ group. *P < 0.05, **P < 0.01 (one-way *ANOVA* test). **(F)** A phagocytosis assay with BMDMs showing exogenous expression of ATP6AP2 inhibited PrCR of SW620 cells and reversed the enhanced PrCR of ATP6AP2^KD^ cells. *P < 0.05, **P < 0.01 (*t* test).

To evaluate whether targeting V-ATPase can be a widely applicable strategy in inducing PrCR, we then assessed the effects of blocking V-ATP activity by pharmacological inhibitors to inhibit its ATPase function in a wider range of human cancer cells including lymphoma, colon cancer and breast cancer (**Figures 3A–D**). Different concentrations of concanamycin and macrophage models including both BMDMs and human peripheral blood monocyte-derived macrophages were used for assessing the role of V-ATPase in regulating PrCR (**Figures 3A–D** and **Figures S2A, B**). Enhanced elimination of cancer cells by macrophages was observed across all the cancer lines examined, when V-ATPase was inhibited. The blockade of the CD47-SIRPα axis further enhanced PrCR induced by V-ATPase disruption (**Figures 3A–D**), suggesting that the V-ATPase-mediated anti-PrCR effect is independent of the inhibitory signaling transduced by CD47. ATPase H+ Transporting Accessory Protein 2, or ATP6AP2, is a critical component that is essential for the proper assembly and function of the V-ATPase complex (57, 58). We then moved to assess the effects of knocking down ATP6AP2 in a variety of cancer cells, and found a similar enhancement of PrCR (**Figure 3E**). Consistently, exogenous expression of ATP6AP2 led to an inhibition of PrCR and was able to reverse the enhanced PrCR due to ATP6AP2 suppression (**Figure 3F** and **Figure S3A**). Our results suggested that the anti-PrCR effects mediated by V-ATPase on cancer cells was in fact a general mechanism. That is, targeting V-ATPase, either by its subunits or assembly machinery, is sufficient to elicit PrCR.

**Figure 4:**
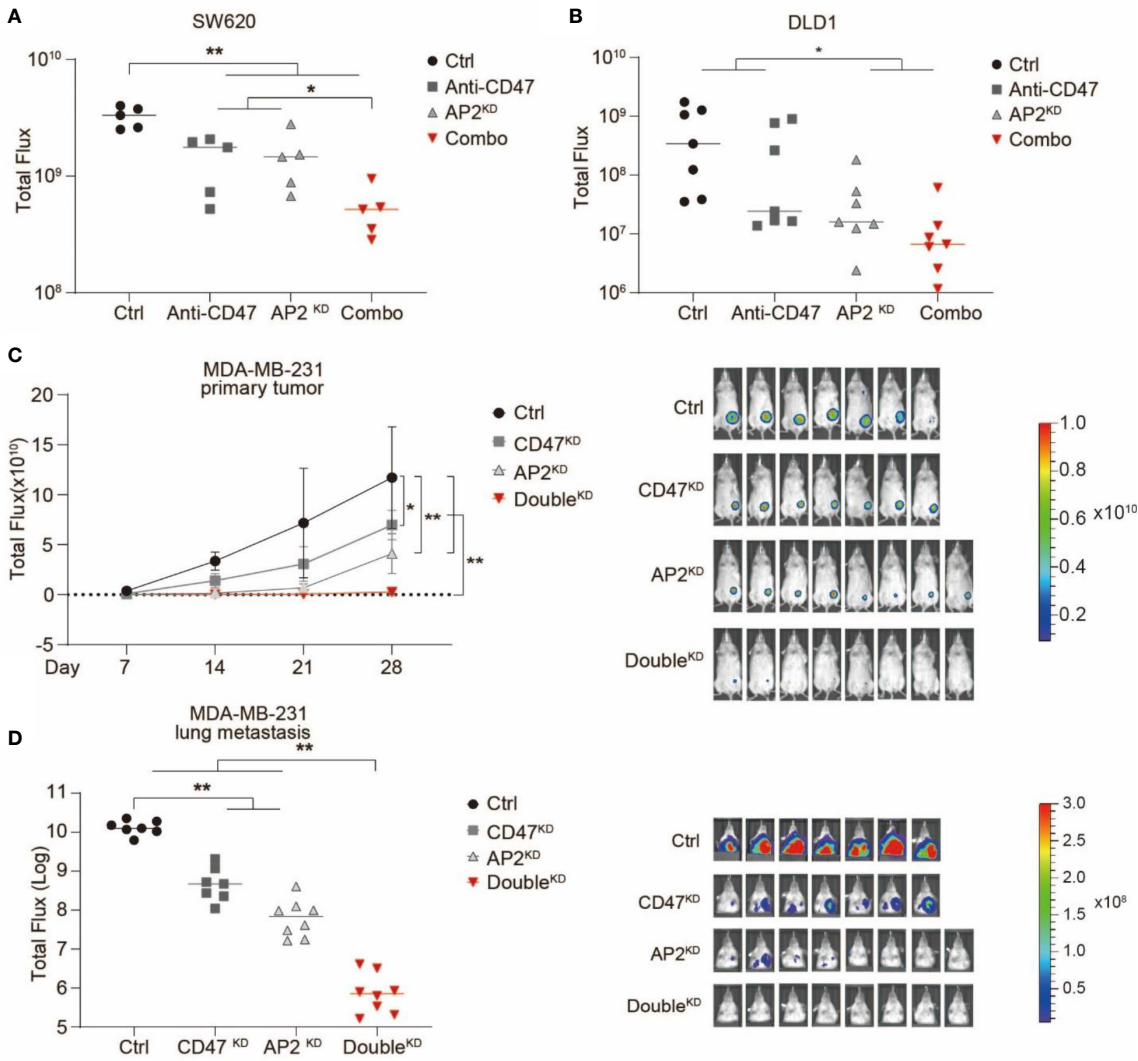
Suppression of ATP6AP2 expression inhibited tumor growth and metastasis in *in vivo* mouse models and synergized with CD47 blockade to yield a dramatic anticancer effect. **(A, B)** Growth of tumors developed by SW620 cells **(A)** and DLD1 **(B)** in RAG2^−/−^ γc^−/−^ mice. Mice engrafted with Ctrl^KD^ cells or ATP6AP2^KD^ cells were treated with PBS or anti-CD47 antibody. Tumor growth was measured by bioluminescence imaging. (n=5 for SW620 model and n=7 for DLD1 model, at 21 days after tumor engraftment). *P < 0.05, **P < 0.01 (*t* test). **(C, D)** Growth **(C)** and metastasis **(D)** of tumors developed by MDA-MB-231 cells in RAG2^−/−^ γc^−/−^ mice. Mice orthotopically engrafted with Ctrl^KD^, CD47^KD^, AP2^KD^, or double knockdown (Double^KD^) MDA-MB-231 cells. Tumor growth and metastasis were measured by bioluminescence imaging (n=7, 7, 8, 8). In **(C)** Left, Tumor growth curve, *P < 0.05, **P < 0.01 (*t* test); Right, animal image from different groups. In **(D)** Left, luminescence signals of lung metastases at day 35, **P < 0.01 (*t* test); Right, animal image from different groups.

Next, we examined the anti-cancer effects of blocking V-ATPase in *in vivo* xenotransplantation models. Human colon cancer lines SW620 or DLD1 cells were transduced with lentiviruses expressing a GFP-luciferase fusion protein. Cas9 and non-targeting or ATP6AP2-targeting sgRNAs were transduced into these cells to establish Ctrl^KD^ or ATP6AP2^KD^ stable cell lines, respectively (**Figure S3B**). RAG2^-/-^, γc^-/-^ or NSG mice (deficient in T, B and NK cells but maintain functional phagocytes) were used for tumor engraftment. After engrafted with Ctrl^KD^ or ATP6AP2^KD^ SW620 or DLD1 cells, the mice were treated with a monoclonal antibody blocking CD47-SIRPα interaction, and tumor development was quantified *via* bioluminescence imaging. We showed that ATP6AP2 knockdown or CD47 blockade alone inhibited tumorigenicity of SW620 and DLD1 in mice, and a combination of ATP6AP2 knockdown and CD47 blockade elicited a strong inhibition of tumor growth and development (**Figures 4A, B** **)**. Next, we used a xenotransplantation breast cancer model to examine the effects of blocking V-ATPase on breast tumor development and metastasis. A human breast cancer line MDA-MB-231 stable lines with Ctrl^KD^, CD47^KD^, ATP6AP2^KD^, or a double knockdown were established by CRISPR/Cas9 (**Figure S2B**) and injected into the mammary fat pad of RAG2^-/-^, γc^-/-^ mice (**Figures 4C, D** **)**. Tumor development and lung metastasis were monitored by bioluminescence imaging. Suppression of either CD47 or ATP6AP2 alone in MDA-MB-231 cells demonstrated anti-cancer effects, and a double knockdown revealed a synergistic effect and dramatically inhibited tumor development and lung metastasis (**Figures 4C, D** **)**.

**Figure 5:**
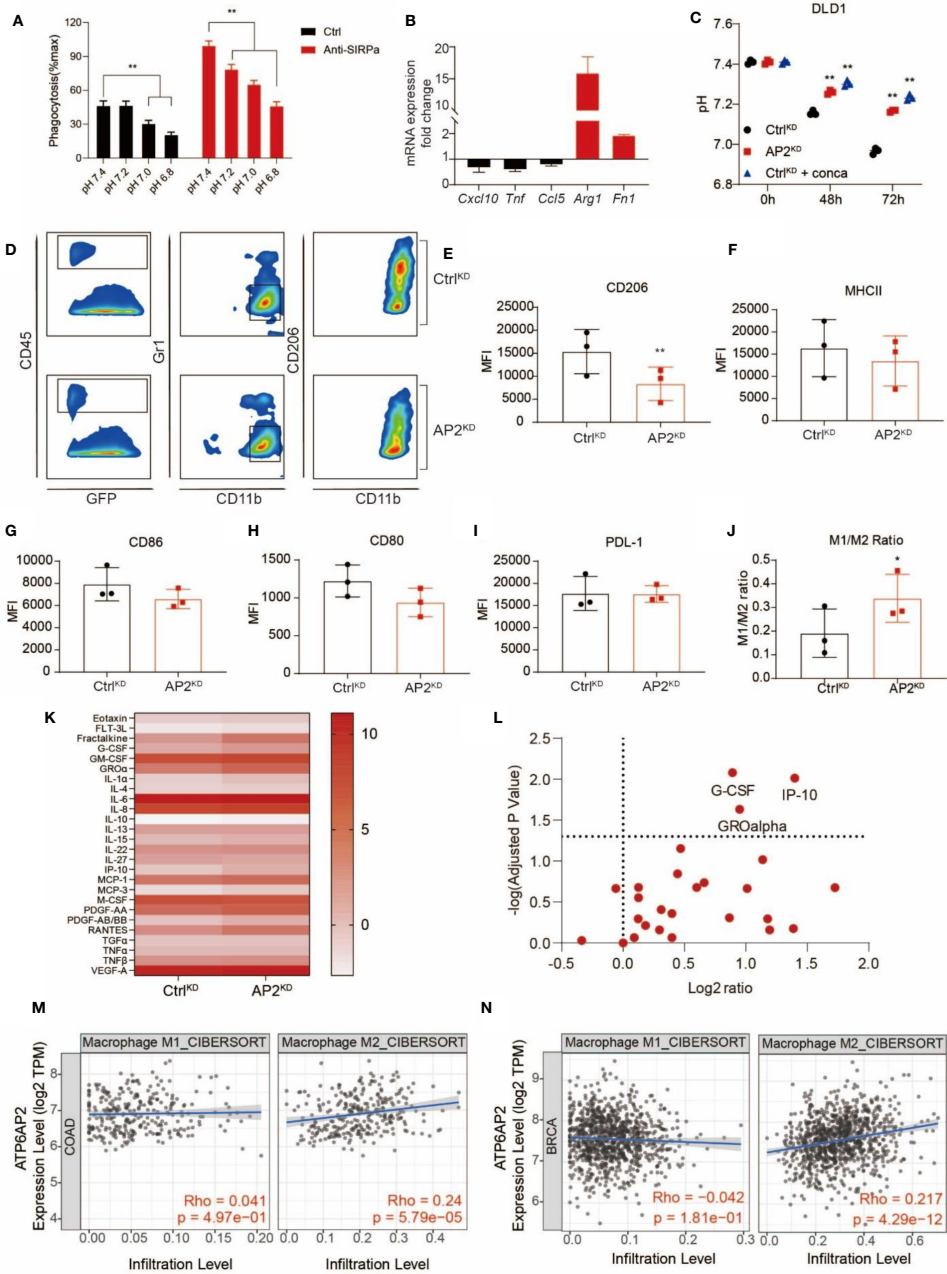
V-ATPase-mediated extracellular acidification facilitated cancer cell evasion of PrCR *via* inhibition of macrophage phagocytic ability and recruitment of M2-like TAMs. **(A)** A phagocytosis assay with BMDMs and SW620 cells in media with a pH ranging from 6.8 to 7.4 showing that acidic medium attenuated macrophage-mediated PrCR toward cancer cells in the absence or presence of CD47 blockades. Each group was compared with corresponding Ctrl group. **P < 0.01 (one-way *ANOVA* test). **(B)** mRNA expression of *Cxcl10, Tnf, Ccl5, Arg1* and *Fn1* in BMDMs cultured at acidic pH as compared to that in BMDMs cultured at natural pH. **(C)** V-ATPase inhibition by ATP6AP2 KD or concanamycin A reverted extracellular acidification by DLD1 cells. Each group was compared with the Ctrl^KD^ group. **P < 0.01 (one-way *ANOVA* test). **(D)** Representative FACS plots showing the expression of CD206 (bottom) on TAMs from tumors developed by Ctrl^KD^ or ATPAP2^KD^ DLD1 cells in mice. **(E–I)** Mean Fluorescent Intensity (MFI) of CD206, MHC II, CD86, CD80 or PD-L1 expression on TAMs from tumors developed by Ctrl^KD^ or ATPAP2^KD^ DLD1 cells in mice. **P < 0.01 (Paired *t* test). **(J)** The ratio of M1:M2 TAMs from tumors developed by Ctrl^KD^ or ATPAP2^KD^ DLD1 cells in mice. *P < 0.05 (Paired *t* test). **(K)** Measurement of cytokine and chemokine secretion profiling of Ctrl^KD^ and ATP6AP2 ^KD^ MDA-MB-231 cells. Scale bars indicated Log_2_(pg/mL). **(L)** Volcano plot showing the differences of cytokine and chemokine secretion by Ctrl^KD^ and ATP6AP2 ^KD^ MDA-MB-231 cells. **(M, N)** Correlation of ATP6AP2 expression with M1 and M2 macrophage infiltration in TCGA colon cancer (n=458) **(M)** and breast cancer (n=1100) **(N)** datasets.

Next, we sought to understand the underlying mechanisms by which V-ATPase in cancer cells confers the ability to escape from PrCR. The extracellular acidification resulting from the Warburg effect leads to an acidic tumor microenvironment with a pH value being as low as 5.8–6.1 (59–62). To investigate the effects of acidic media on PrCR, we performed a phagocytosis assay in media with a pH ranging from 5.8 to 8.8 titrated by lactic acid, and found that cancer cell phagocytosis was dramatically inhibited in the acidic medium compared to the neutral or alkaline medium (**Figure 5A** and **Figure S4A**). The effects of blocking V-ATPase in promoting PrCR were diminished when the extracellular media were maintained acidic by excessive lactic acid (**Figure S4B**). While both M1- and M2-like macrophage respond to blockade of CD47-Sirpα axis to carry out PrCR, stronger phagocytic ability has been associated with M1-polarized macrophages (63). We demonstrated when macrophages were cultured in acidic media, the expression of markers for M1-like states (64, 65) including *Cxcl10, Tnf* and *Ccl5* was downregulated, whereas the expression of key markers for M2-like macrophages (64, 65) including *Arg1* and *Fn1* was dramatically upregulated, in macrophages cultured in acidic media (**Figure 5B**). Next, we assessed the effects of acidic microenvironment in the recruitment and polarization of TAMs in the tumors and investigated whether targeting V-ATPase could reverse these effects and generate a pro-phagocytic microenvironment. We demonstrated that disruption of V-ATPase reverted extracellular pH back to a neutral level (**Figure 5C** and **Figures S4C, D**) without directly impacting the viability of cancer cells (**Figures S4E, F**). Therefore, we engrafted mice with Ctrl^KD^ DLD1 cells or ATP6AP2^KD^ DLD1 cells which are deficient in the capability of acidifying extracellular medium, and assessed TAMs from tumors developed by these cells by examining their cell surface marker expression. While the expression of markers related to antigen-presentation, such as CD80 and CD86 (66), as well as ligands for immune checkpoints, such as PD-L1, remained unchanged, there was a significant downregulation of CD206 expression in macrophages taken from tumors resulted from ATP6AP2^KD^ cancer cells (**Figures 5D–I**). Because CD206 is a well-defined marker representing polarization toward M2-like macrophages (67), we then examined the M1- (classically activated macrophages; CD206^-^, MHC II^high^) and M2- (alternatively activated macrophages; CD206^+^, MHC II^low/neg^) like tumor-associated macrophages, and found a significant increase of the ratio of M1:M2 macrophages (**Figures 5J**), suggesting that the disruption of the acidic tumor microenvironment had compromised the recruitment and enrichment of CD206-expressing M2-like TAMs in the tumors. We further investigated the cytokine and chemokine profiling of ATP6AP2 ^KD^ MDA-MB-231 cells by quantifying 48 different human cytokine and chemokine secreted by MDA-MB-231 cells at the protein level. We found significantly higher G-CSF, IP-10 and GRO*α* concentration in the ATP6AP2 ^KD^ cell medium (**Figures 5K, L** **)**. Although the roles of G-CSF in macrophage polarization remained to be determined, IP-10 and GRO*α* has been found in many studies to be involved in recruiting proinflammatory leukocytes and reprograming macrophage towards M1 polarization (68, 69). These results suggested that the inhibition of V-ATPase function in cancer cells may be linked to the production in proinflammatory cytokines, although such a link to eliciting the tumor microenvironment toward a proinflammatory state remains to be determined by further analysis. In addition, we found V-ATPase related genes including ATP6V1A, ATP6V0D1, ATP6AP1 and ATP6AP2 expression were positively correlated with M2 macrophage infiltration but not M1 macrophage in TCGA colon cancer and breast cancer dataset (**Figures 5M, N** and **Figures S4G–S4L**), which is consistent with our findings that acidic tumor microenvironment facilitated polarization and recruitment of M2-like macrophages.

**Figure 6:**
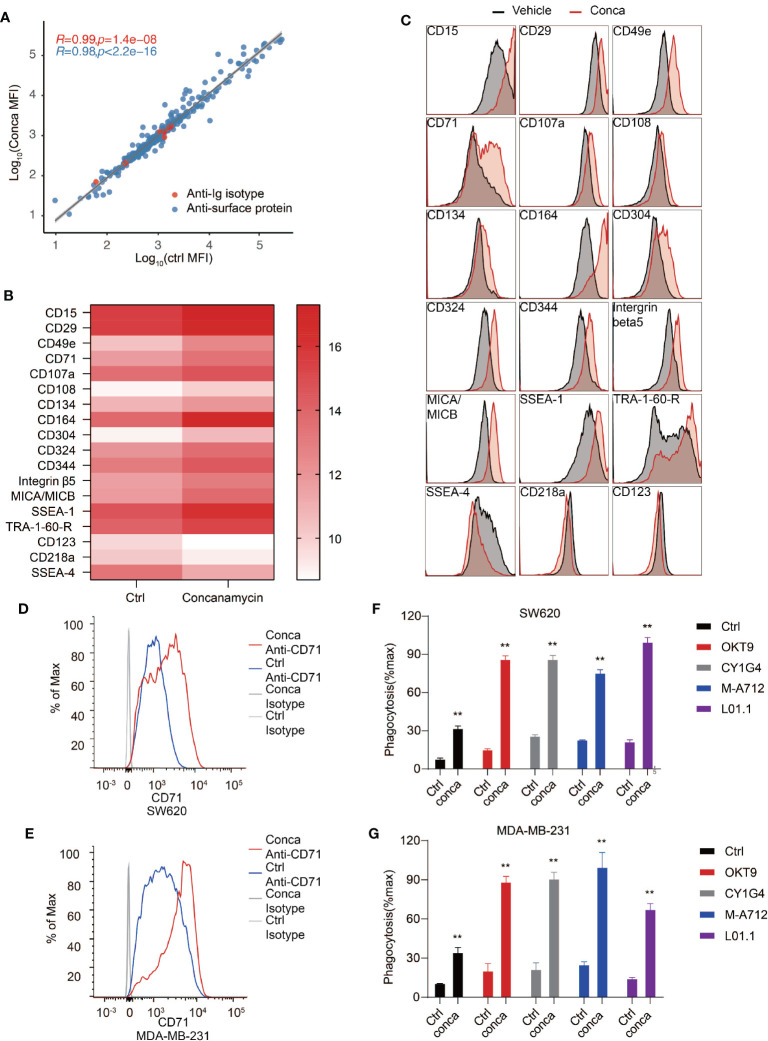
Cell surface proteome analysis revealed CD71 as a potential target for synthetic PrCR upon V-ATPase inhibition. **(A)** Examination of cell surface marker expression on SW620 cells treated with vehicles or concanamycin with an antibody array containing a panel of 332 antibodies. **(B)** Identification of 15 upregulated and 3 downregulated surface proteins upon concanamycin treatment. **(C)** Representative flow cytometry staining of the upregulated and downregulated surface proteins on SW620 upon concanamycin treatment. **(D, E)** Flow cytometry analyses showing the upregulation CD71 expression on the cell surface of concanamycin-treated SW620 **(D)** or MDA-MB-231 **(E)** cells. **(F, G)** Phagocytosis assay with BMDMs showing that concanamycin treatment enabled macrophage-mediated PrCR of SW620 **(F)** or MDA-MB-231 **(G)** cells induced by CD71 antibodies. Each group was compared with corresponding Ctrl group. **P < 0.01 (*t* test).

Cell surface proteome is the interface mediating intercellular interaction including communications between cancer cells and the immune system, and many of the cell surface receptors have become important therapeutic targets for blocking cancer cell proliferation, survival and immune evasion (70–73). Therefore, we investigated the effects of V-ATPase disruption on cancer cell surface proteomic landscape. SW620 cells were treated with vehicles or concanamycin to inhibit V-ATPase activity and subjected to an antibody array to examine cell surface marker expression. A panel of 332 antibodies were included in the antibody array and cell surface marker expression was assessed by flow cytometry. We discovered that 15 proteins were significantly upregulated on the cell surface, and 3 proteins were significantly downregulated (**Figures 6A–C**). CD71 is one such protein whose expression on SW620 cells was dramatically upregulated upon blockade of V-ATPase (**Figures 6A–C**). CD71, or transferrin receptor 1, was previously identified as a therapeutic target for antibody-mediated cancer therapy (45, 74). The anti-cancer effects of CD71 antibodies can be achieved by direct cytotoxic effect through crosslinking of the receptor, or by antibody-dependent cell-mediated cytotoxicity/phagocytosis (ADCC/ADCP) based on Fc effector functions. We confirmed induction of CD71 surface exposure upon V-ATPase blockade in both SW620 and MDA-MB-231 cells (**Figures 6D, E** **)** and reasoned that the upregulated exposure of CD71 induced by V-ATPase blockade may further enhance the efficacy of anti-CD71 antibodies in inducing PrCR. Indeed, we found that treatment of SW620 or MDA-MB-231 cells with anti-CD71 antibodies was able to induce PrCR of the cells in a Fc-dependent manner (**Figures 6F, G** **)**. Furthermore, using four different monoclonal antibodies recognizing CD71, we demonstrated that cells with their V-ATPase blocked had become significantly more susceptible to CD71 antibody-induced PrCR (**Figures 6F, G** **)**.

## Discussion

The identification of T cell immune checkpoints and the development of therapies targeting these immune inhibitory regulatory mechanisms have revolutionized cancer therapeutics (75, 76). As essential components of immune regulatory programs, negative signaling induced by ligand binding causes a downregulation of the immune response to avoid overactivation of immune activities (77). So far, over ten of such immune checkpoints have been identified, such as PD-1, CTLA4, TIM3, and so on. These immune checkpoints have been found to function at different stages of the T cell’s lifecycle, including the priming, proliferation, and effector phases. Blocking immune checkpoints has been found to elicit T and NK cell attack of malignant cells and lead to significant anti-cancer immune responses (78). Tumor-associated macrophages represent the largest population of immune cells present in tumors and metastases (4, 5). The identification of immune checkpoints on TAMs and their ligands on cancer cells revealed the therapeutic potential for re-activating macrophages in the tumor microenvironment for direct phagocytosis and elimination of cancer cells (10, 11). The blockade of immune checkpoints on macrophages subsequently restored their ability to carry out PrCR. Recent progress in numerous preclinical models and clinical trials has revealed the therapeutic potential of inducing PrCR as a new class of promising immunotherapy (21, 22, 53).

Despite the exciting progress, it remains unclear whether and how the tumor microenvironment at different stages of tumor development impacts the PrCR ability of TAMs. The metabolic programs in cancer cells are characterized by aerobic glycolysis even when sufficient oxygen is present, known as the Warburg effect, during which 2 mol of ATP can be generated per mol of glucose (32). This directly contrasts with oxidative phosphorylation, in which 32 mol of ATP can be generated per mol of glucose. Glucose is converted to lactic acid during aerobic glycolysis, which is secreted out of the cell to acidify the microenvironment (40). The Warburg effect allows for rapid ATP synthesis and provides intermediate biosynthetic materials, both of which are critical for meeting the high proliferative demands of cancer cells (33, 43). In addition to this, acidity generated by lactic acid secretion into the extracellular medium leads to remodeling at the tumor-stroma interface to facilitate tumor invasion (79). The accumulation of lactic acid and acidification of tumor sites have been reported to block lymphocyte infiltration into tumors by limiting glucose supply and diminishing T cell and NK cell activity (80, 81).

Here we show that the acidic microenvironment resulting from the Warburg effect significantly attenuates macrophage-mediated PrCR against cancer cells. Interestingly, in addition to directly inhibiting the phagocytic ability of macrophages, an acidic microenvironment may be involved in polarizing TAMs towards a M2-like phenotype, therefore compromising the detection and recognition of cancer cells. We demonstrate that targeting V-ATPase on cancer cells to disrupt their proton pumping machinery dramatically promoted cancer cell recognition and PrCR. Importantly, resistance to PrCR induced by CD47-blockade has been described in several types of cancers, which is not attributable to the deficiency of CD47 expression on these cells nor the contribution of other known “don’t eat me” signals, suggesting the existence of additional anti-PrCR mechanisms (82–84). Our results reveal a strategy of activating PrCR independent of and complementary to current PrCR-based therapies by blocking CD47 on cancer cells. Therefore, this may represent a widely applicable treatment approach for various cancers, including those with limited or no CD47 expression. Cell surface proteins on cancer cells play important roles in transmitting extracellular signals and mediating their interactions with the immune cells, and thus are critical for cancer cells to maintain a highly proliferative state and escape from immunosurveillance (70–73). Therefore, cell surface receptors are important therapeutic targets for cancer treatment. In this study, we deduced a “synthetic PrCR” model, in which the blockade of V-ATPase enhances cell surface exposure of therapeutic markers such as CD71 and the ADCP effects from targeting such markers using antibodies was found to be dramatically improved. Future studies will be performed to further characterize subgroups of TAMs and decipher their molecular phenotypes in the acidic tumor microenvironment. This will then be used to develop strategies to convert TAM populations with a hampered PrCR ability to PrCR-competent groups for superior success at cancer detection and subsequent elimination.

## Data Availability Statement

The raw data supporting the conclusions of this article will be made available by the authors, without undue reservation.

## Ethics Statement

The animal study was reviewed and approved by Laboratory Animal Care at City of Hope Comprehensive Cancer Center.

## Author Contributions

JC, XC, BL and MF designed the research, and performed and analyzed the experiments; ZZ, SC, SM, GN and SL performed and analyzed the experiments; LW, WG, JY and PL contributed to the experimental design and scientific input; JC and XC prepared the figures; JC, XC, SL and MF wrote and edited the manuscript; MF supervised the laboratory. All authors contributed to the article and approved the submitted version.

## Conflict of Interest

MF declares patent applications pertaining to stimulating TLR/BTK signaling to promote CRT in macrophages assigned to the Stanford University and equity and/or consulting with Forty Seven, Inc.

The remaining authors declare that the research was conducted in the absence of any commercial or financial relationships that could be construed as a potential conflict of interest.
