# Supplementary material for: Warburg Effect Is a Cancer Immune Evasion Mechanism Against Macrophage Immunosurveillance
Source: Front Immunol. 2021 Feb 2;11:621757. doi: 10.3389/fimmu.2020.621757 (PMC7884830; doi:10.3389/fimmu.2020.621757)
Supplement: Supplementary file 1 [file DataSheet_1.pdf]

Figure S1

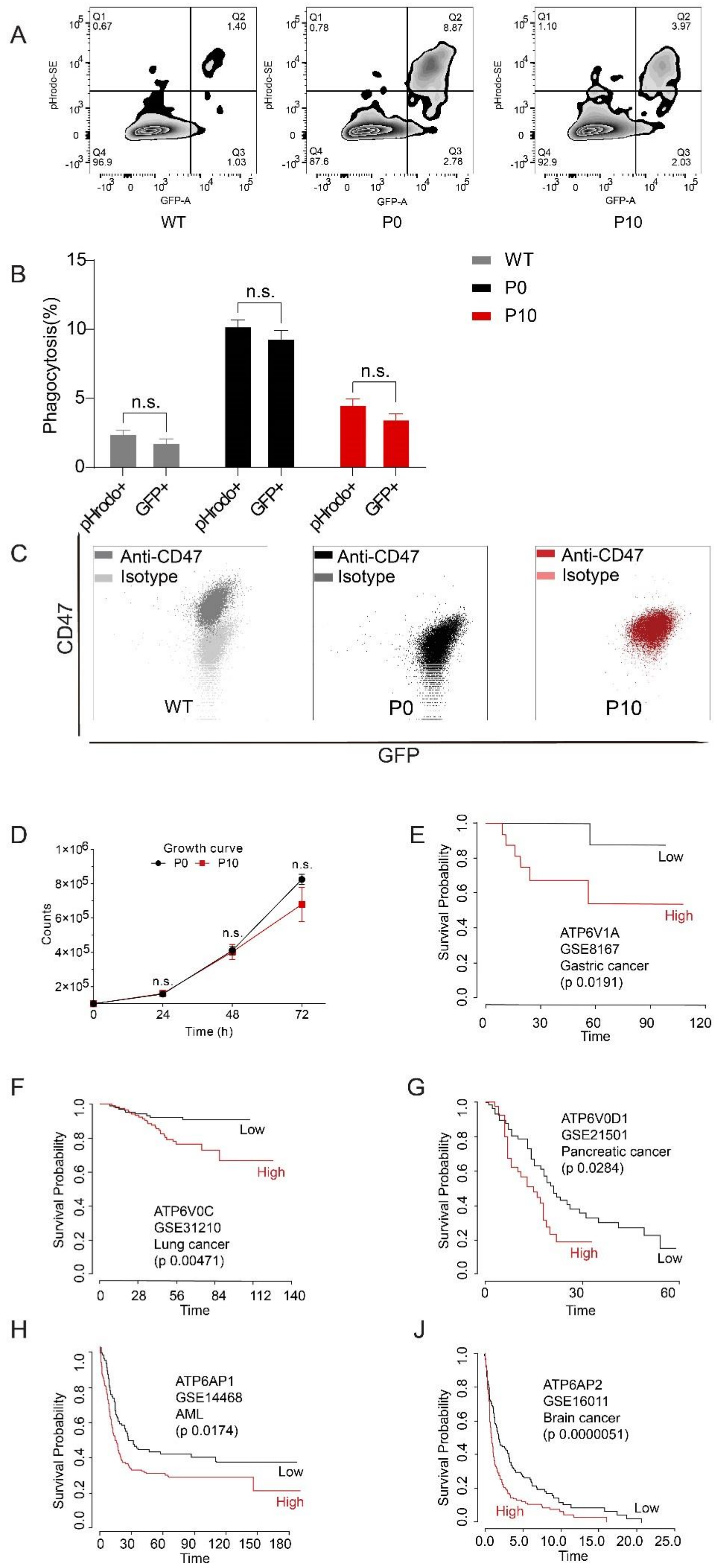
A
Q1 0.67
Q2 1.40
Q4 96.9
Q3 1.03
pHrodo-SE
GFP-A
WT
Q1 0.78
Q2 8.87
Q4 87.6
Q3 2.78
P0
Q1 1.10
Q2 3.97
Q4 92.9
Q3 2.03
P10
B
Phagocytosis(%)
n.s.
WT
P0
P10
pHrodo+
GFP+
C
Anti-CD47
Isotype
CD47
GFP
WT
P0
P10
D
Growth curve
P0
P10
Counts
Time (h)
n.s.
E
Survival Probability
Low
High
ATP6V1A
GSE8167
Gastric cancer
(p 0.0191)
Time
F
Low
High
ATP6V0C
GSE31210
Lung cancer
(p 0.00471)
Time
G
ATP6V0D1
GSE21501
Pancreatic cancer
(p 0.0284)
High
Low
Time
H
ATP6AP1
GSE14468
AML
(p 0.0174)
Low
High
Time
J
ATP6AP2
GSE16011
Brain cancer
(p 0.0000051)
High
Low
Time

Figure S1

(A) and (B) A phagocytosis assay with BMDMs and SW620 WT, CD47^{KO} P0 or CD47^{KO} P10 cells. SW620 cells were GFP^{+} and were labeled with pHrodo red dye prior to coculture with BMDMs. pHrodo dye was non-fluorescent outside the cells and became red (PE^{+}) in phagosomes, indicating engulfment of SW620 cells by macrophages. After phagocytosis, cells were analyzed by flow cytometry and macrophages (F4/80^{+}) were gated and shown in the FACS plots (A). The bar graphs (B) showed the phagocytosis index, quantified as the number of macrophages that have phagocytosed SW620 cells (GFP+ or PE+) divided by the number of total macrophages. Quantification of phagocytosis based on GFP or pHrodo (PE) showed no difference. (*t* test)

(C) Flow cytometry analysis of CD47 expression on SW620 WT, P0 and P10 cells in dot plots.

(D) Measurement of the proliferation rates of SW620 CD47^{KO} P0 and P10 cells. P10 cells showed no change of proliferation compared to P0 cells, analyzed by counting cells at indicated time points. (*t* test)

(E-J) Upregulated ATP6V1A expression was correlated with a worse overall survival in gastric cancer patients(E). Upregulated ATP6V0C expression was correlated with a worse overall survival in lung cancer patients(F). Upregulated ATP6V0D1 expression was correlated with a worse overall survival in pancreatic cancer patients(G). Upregulated ATP6AP1 expression was correlated with a worse overall survival in AML patients(H). Upregulated ATP6AP2 expression was correlated with a worse overall survival in brain cancer patients(J).

Figure S2

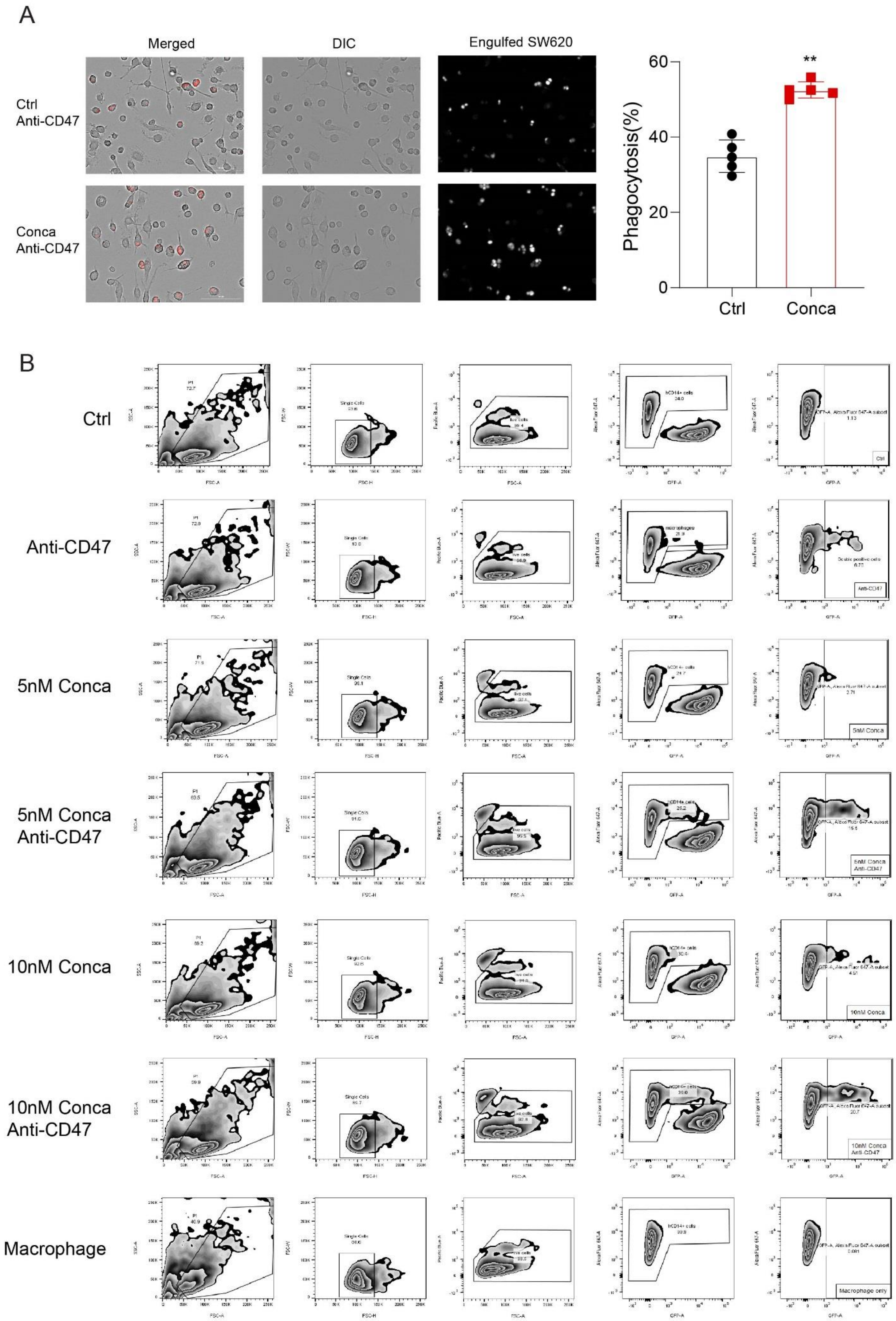

A
Merged
DIC
Engulfed SW620
Ctrl
Anti-CD47
Conca
Anti-CD47
Phagocytosis(%)
**
60
40
20
0
Ctrl
Conca
B
Ctrl
Anti-CD47
5nM Conca
5nM Conca
Anti-CD47
10nM Conca
10nM Conca
Anti-CD47
Macrophage

Figure S2

(A) A microscopy-based phagocytosis assay with BMDMs and SW620 cells showing that pharmacological inhibition of V-ATPase with concanamycin A promoted PrCR. Left, representative microscopy images of BMDMs with concanamycin-treated SW620 cells, in the presence of anti-CD47 antibody. SW620 cells were labeled with pHrodo red dye prior to coculture with BMDMs. PHrodo dye was non-fluorescent outside the cells and became red in phagosomes, indicating engulfment of SW620 cells by macrophages. Right, quantification of phagocytosis, showing as the numbers of macrophages with red dots inside (macrophages that have phagocytosed SW620 cells) divided by the numbers of total macrophages. $^{**}P<0.01$ (*t* test).

(B) Gating strategy for Figure 3C. $CD14^{+}$ macrophages were gated for calculating phagocytosis.

Figure S3

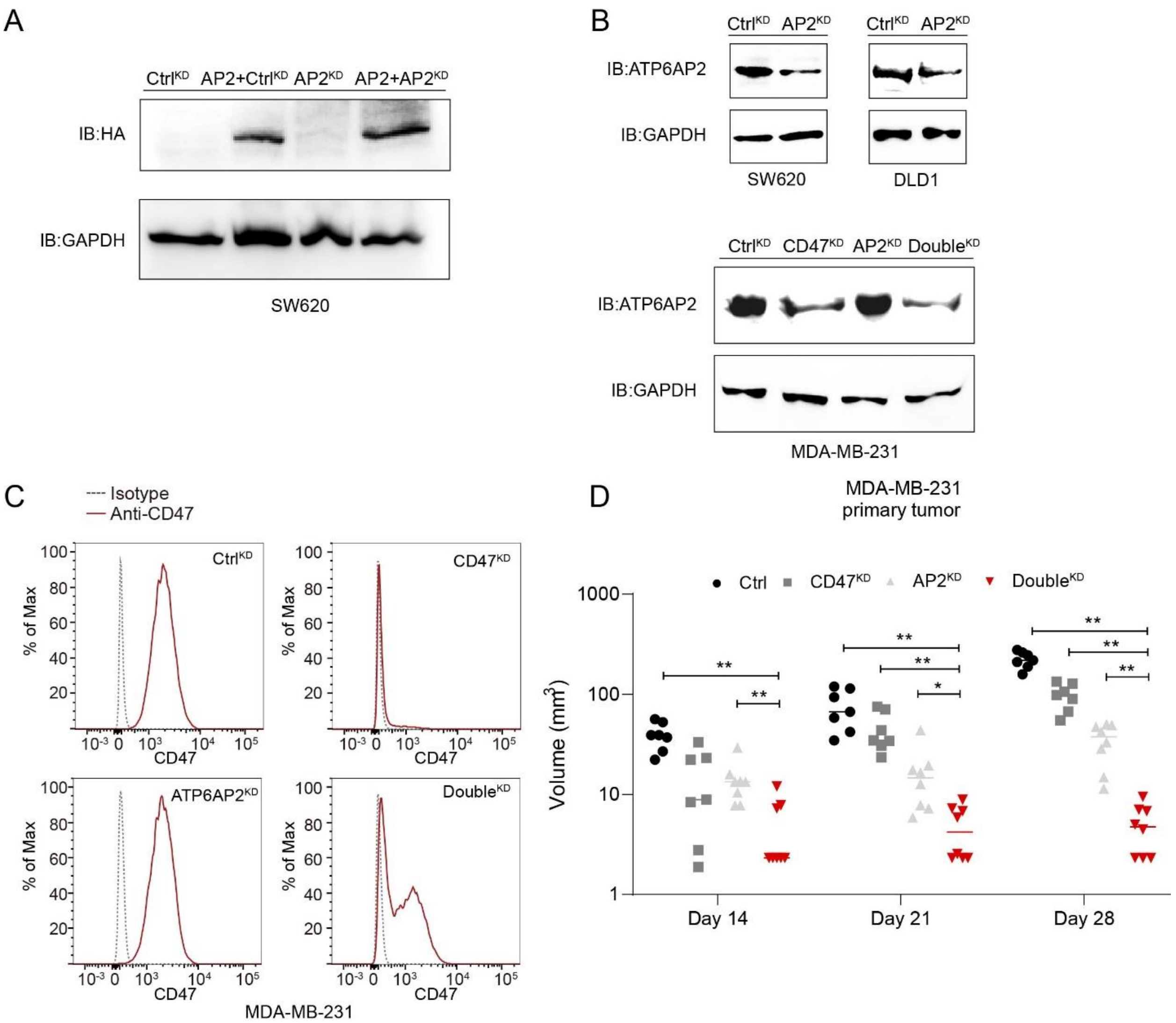

Figure S3

(A) Immunoblotting showing the exogenous expression of ATP6AP2 in SW620 cells, probed with anti-HA antibody.

(B) Immunoblotting showing the expression of ATP6AP2 in Ctrl^{KD}, CD47^{KD}, AP2^{KD} or Double^{KD} SW620, DLD1 or MDA-MB-231 cells, probed with anti-ATP6AP2 antibody and anti-GAPDH antibody (loading control).

(C) Flow cytometry analysis showing the expression of CD47 in Ctrl^{KD}, CD47^{KD}, AP2^{KD} or Double^{KD} MDA-MB-231 cells.

(D) Tumor size of mice orthotopically engrafted with Ctrl^{KD}, CD47^{KD}, AP2^{KD}, or double knockdown (Double^{KD}) MDA-MB-231 cells at day7, day14 and day 28. *P<0.05, **P<0.01 (t test).

Figure S4

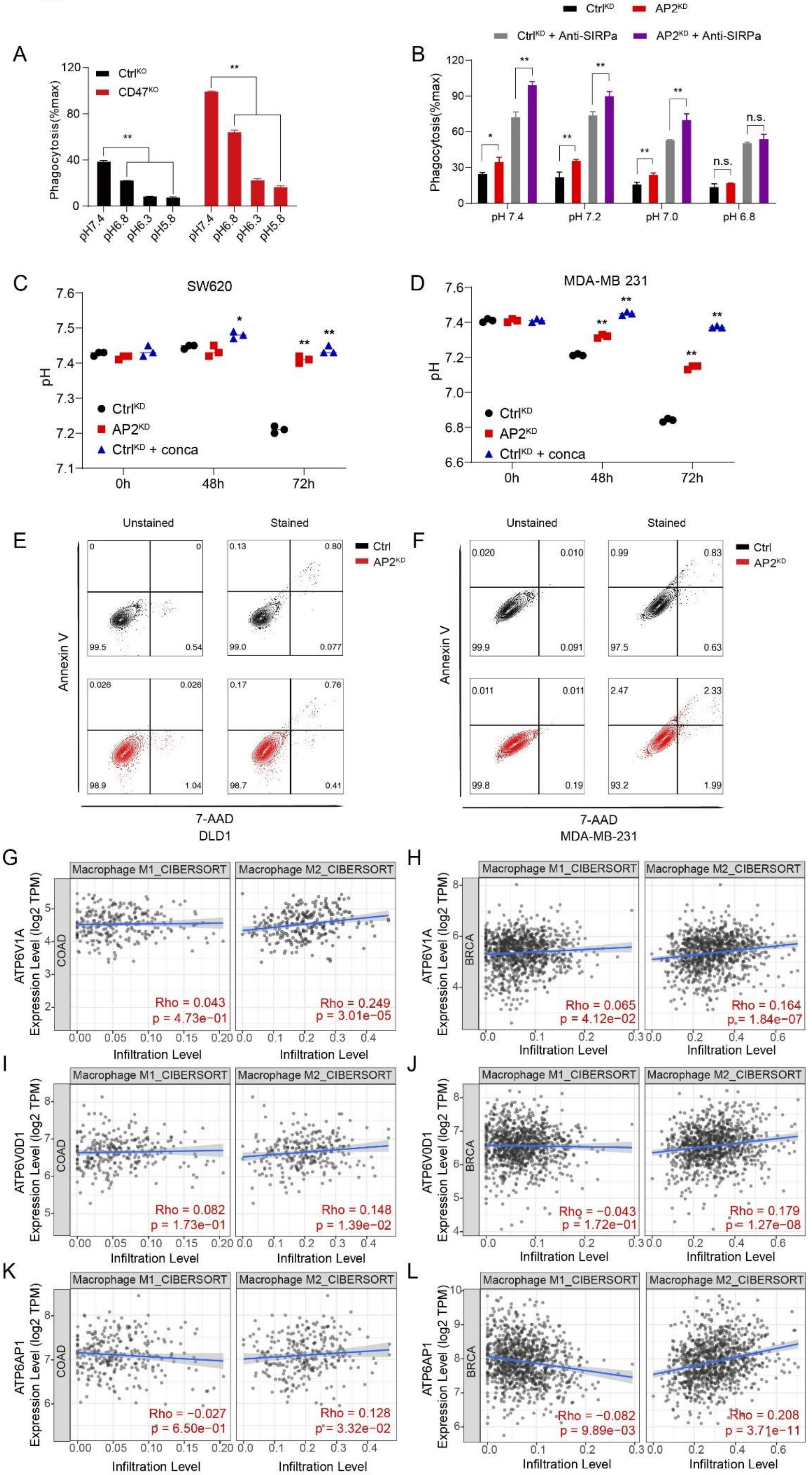
A
Ctrl^KO
CD47^KO
Phagocytosis(%max)
pH7.4
pH6.8
pH6.3
pH5.8
B
Ctrl^KD
AP2^KD
Ctrl^KD + Anti-SIRPa
AP2^KD + Anti-SIRPa
pH 7.4
pH 7.2
pH 7.0
pH 6.8
n.s.
C
SW620
pH
Ctrl^KD
AP2^KD
Ctrl^KD + conca
0h
48h
72h
D
MDA-MB 231
E
Unstained
Stained
Ctrl
AP2^KD
Annexin V
7-AAD
DLD1
F
MDA-MB-231
G
H
I
J
K
L
Macrophage M1_CIBERSORT
Macrophage M2_CIBERSORT
ATP6V1A
ATP6V0D1
ATP6AP1
Expression Level (log2 TPM)
COAD
BRCA
Infiltration Level
Rho = 0.043
p = 4.73e-01
Rho = 0.249
p = 3.01e-05
Rho = 0.065
p = 4.12e-02
Rho = 0.164
p = 1.84e-07
Rho = 0.082
p = 1.73e-01
Rho = 0.148
p = 1.39e-02
Rho = −0.043
p = 1.72e-01
Rho = 0.179
p = 1.27e-08
Rho = −0.027
p = 6.50e-01
Rho = 0.128
p = 3.32e-02
Rho = −0.082
p = 9.89e-03
Rho = 0.208
p = 3.71e-11

Figure S4

(A) A phagocytosis assay with BMDMs and SW620 cells in media with a pH ranging from 5.8 to 7.4 showing that acidic medium attenuated macrophage-mediated PrCR toward cancer cells in the absence or presence of CD47 blockades. Each group was compared with corresponding Ctrl group. ** $P<0.01$ (one-way *ANOVA* test).

(B) Acidification by adding extra lactic acid could reverse the ATP6AP2 ^{KD} promoted PrCR in the absence or presence of CD47/SIRPa blockade. Each group was compared with Ctrl^{KD}. * $P<0.05$, ** $P<0.01$ (*t* test)

(C-D) Inhibition of V-ATPase function by ATP6AP2^{KD} or concanamycin A reverted extracellular acidification by SW620 cells (A) and MDA-MB-231 cells (B). Each group was compared with the Ctrl^{KD} group. * $P<0.05$, ** $P<0.01$ (one-way *ANOVA* test).

(E-F) Measurement of cell viability. Ctrl^{KD} and ATP6AP2^{KD} DLD1 and MDA-MB-231 cells were stained by Annexin V and 7-AAD and analyzed by flow cytometry. Similar percentage of the Ctrl KD or AP2 KD cells stained negative for AnnexinV and/or 7-AAD, suggesting that knockdown of ATP6AP2 showed no direct impact on cell viability.

(G-L)Positive correlation of V-ATPase related genes including V1A (G and H), V0D1 (I and J) and AP1 (K and L) expression with M2-like but not M1-like tumor-associated macrophage infiltration in colon cancer and breast cancer tumors from TCGA datasets, by TIMER 2.0 analysis.
